# Reliable variability in subjective responses to parenteral hydromorphone administration: empirical confirmation of an opioid non-responder phenotype

**DOI:** 10.1038/s41386-025-02271-4

**Published:** 2025-11-18

**Authors:** Jennifer D. Ellis, Eleanor Blair Towers, Kelly E. Dunn, Siny Tsang, Traci Speed, Sheera F. Lerman, Michelle Mei, Wendy Lynch, Eric C. Strain, Michael T. Smith, Patrick H. Finan

**Affiliations:** 1 Department of Psychiatry and Behavioral Sciences, Johns Hopkins University School of Medicine, Baltimore, USA; 2 Department of Anesthesiology, University of Virginia School of Medicine, Charlottesville, USA; 3 Kahlert Institute for Addiction Medicine, University of Maryland, Baltimore, MD USA; 4 Department of Psychiatry and Neurobehavioral Sciences, University of Virginia, School of Medicine, Charlottesville, USA

**Keywords:** Addiction, Risk factors

## Abstract

A subset of individuals without a history of prolonged or problematic opioid use demonstrate attenuated subjective responses to orally administered opioids despite physiological and analgesic responses. This phenotype may confer elevated risk for greater analgesic requests and physiologic dependence, but it is unclear if this is driven by differences in route of administration. This study evaluated responses to cumulative dosing of parenteral hydromorphone, versus placebo, in persons without a history of significant opioid use to further identify and characterize this subgroup. Individuals without opioid use disorder (*N* = 82) were exposed to a cumulative hydromorphone dosing procedure, during which they completed measures of subjective effects and were assessed for physiological opioid responses. Linear mixed effects regressions were used to examine changes in subjective and physiological measures as a function of responder phenotype, drug condition, and time. Approximately 31.7% of the sample were classified as Opioid Non-Responders. These individuals had attenuated changes in subjective responses to hydromorphone relative to other participants, despite equivalent physiological responses. Race and sex did not predict Opioid Responder status. These findings confirm the presence of a “Opioid Non-Responder” phenotype for the first time in the context of a cumulative, parenteral dosing paradigm. Further research is warranted to elucidate the clinical implications and potential risk or protective factors underlying this phenotype.

## Introduction

Despite efforts to reduce prescribing, opioids are a mainstay for pain management in both inpatient and outpatient settings [1, 2]. Variability in analgesic requirements, subjective responses to opioids, and opioid use behaviors have been documented in clinical settings [3–7]; however, the underlying cause of this variability remains poorly characterized and is often attributed to differences in the underlying pain condition rather than individual-level variability in drug response.

A growing number of empirical studies in individuals without opioid physical dependence suggest that a subset of individuals do not subjectively detect opioid effects at doses that are considered “therapeutic”, and that such persons require higher doses before reporting opioid-like effects, and generally do not report liking opioids more than placebo. For instance, Antoine and colleagues found that 39% of participants with no opioid physical dependence did not rate “drug liking” for 30 mg oxycodone more than 20 points higher than placebo on a visual analogue scale (VAS) measure [8] and also reported significantly lower overall “drug effects”. Despite attenuated responses to multiple subjective measures, this subgroup experienced similar levels of maximum pupillary constriction (a valid metric of opioid agonist activity) as participants who experienced strong drug effects. This is consistent with a separate analysis that found a subset of individuals were unable to subjectively detect oral hydromorphone on a measure of “Drug Effects” despite showing physiological and analgesic responses to hydromorphone [9]. Recently, McKendrick and colleagues found that 30% of healthy individuals with limited or no prior history of opioid exposure did not subjectively endorse clinically relevant “Drug Effects” at low (i.e., 2 mg), moderate (i.e., 4 mg), and high (i.e., 8 mg) doses of oral hydromorphone, and reported attenuated pleasant drug effects on most items (e.g., “energized” or “stimulated”) [10]. However, all study participants demonstrated opioid-specific changes on multiple physiological parameters (pupil diameter, heart rate, and blood oxygenation), as well as experimental measures of acute analgesia (i.e., pain threshold, pain tolerance), indicating that hydromorphone produced expected agonist effects independent of their subjective awareness. Importantly, participants, including those with attenuated sensitivity to the subjective effects of opioids, continued to report subjective pain following laboratory-induced pain tasks, even after receiving the highest dose of hydromorphone and despite objective evidence of analgesia.

Collectively, these findings provide evidence for an underlying “Opioid Non-Responder” phenotype, defined as individuals who exhibit physiological responses to opioids but report minimal or no subjective drug effects, that may have important implications for opioid use trajectories and clinical decision-making. These analyses build on prior research by examining parenteral administration to surmount two key limitations of oral dosing. First, oral opioid effects are subject to significant variability due to differences in first-pass metabolism through the liver [11–14]. Second, sole reliance on data derived from oral administration limits external validity for highly common clinical contexts requiring parenteral administration of an opioid (e.g., cancer care [15] and perioperative anesthesia [16]). Cumulative parenteral dosing in acute pain management can lead to differences in opioid doses given based on individual patient response [17, 18]. In the present study, we utilized a cumulative parenteral dose design in persons with no history of opioid use disorder (OUD) and evaluated whether this phenotype could also be observed following parenterally administered hydromorphone relative to placebo. It was hypothesized that a portion of the sample would not subjectively detect hydromorphone and that hydromorphone injections, relative to placebo, would lead to objective physiological changes (i.e., decreases in pupil size and heart rate) regardless of Responder Status.

## Materials and Methods

### Study design overview

The present analyses include secondary data gathered during the screening periods of two separate studies with harmonized research methods designed to evaluate the effects of experimental sleep disruption on subjective responses to hydromorphone administration (NCT04299490 and NCT03680287). The sleep disruption-related effects will be reported in a separate manuscript. All participants in both studies were required to undergo an opioid challenge session as the final part of the screening process prior to moving into the experimental sleep disruption phase. The present manuscript focuses exclusively on the opioid challenge data.

Study procedures for both studies were approved by the Johns Hopkins Institutional Review Board (IRB), and all participants provided informed consent.

### Participants

Study 1 recruited both healthy adults (*N* = 27) and adults with chronic low back pain (CLBP; *N* = 10), and Study 2 recruited healthy adults (*N* = 45). We included persons with CLBP because they were not presently taking opioids and lacked a prior history with OUD, similar to the healthy participants. A sensitivity analysis that excluded individuals with CLBP showed largely consistent results (see Supplementary Table 1). For both studies, individuals did not have major psychiatric conditions or significant medical conditions other than CLBP, had a BMI < 40, screened negative for recreational drug use, did not have a history of adverse reactions to opioids, were not pregnant, did not have a history of OUD, and did not have clinically significant abnormal lab findings (e.g., complete blood count, metabolic profile, or ECG). More detailed eligibility criteria for each study are presented in Supplementary Materials.

### Screening

For both studies, eligibility was determined via a combination of questionnaires delivered over REDCap, semi-structured clinical interviews using the MINI International Neuropsychiatric Interview [19], history and physical examination, urine and blood tests, and an ECG. These measures included assessment of substance use disorder history; persons who tested positive for illicit substances via urinalysis or who reported recent use were excluded.

### Opioid dosing details

Both Study 1 and Study 2 employed a challenge session that used a double-blind, cumulative hydromorphone dose design characterized by an initial placebo dose and subsequent ascending doses of hydromorphone. Participants were required to provide a negative urine drug sample and a negative pregnancy test prior to starting the session. Participants received a calorie-controlled breakfast before procedures began. Opioid challenge sessions lasted up to 420 min (i.e., in Study 1). Participants remained seated in a study session room during opioid challenge sessions. When not completing study tasks, participants were permitted to read, use their phones, or participate in other low-intensity activities.

Participants and staff were blinded to the doses, ordering of placebo versus hydromorphone administration, and ordering of hydromorphone doses. The purpose of this session was twofold: 1) to determine if participants could differentiate hydromorphone from placebo, and 2) to verify that only participants who tolerated hydromorphone would be enrolled in the parent trials.

#### Study 1 (n = 37)

At study start, an intramuscular (IM) route of administration was used to administer hydromorphone in the following ascending order: [0 (Saline), 0.33, 0.65, and 1.3 mg/ 70 kg], separated by 60-minute inter-injection intervals. Five participants (all healthy) were exposed to this initial dosing scheme. Due to the emergence of adverse events (e.g., unpredictable and prolonged periods of vomiting) related to hydromorphone in two participants, we lowered the overall cumulative dose to increase safety, as advised by the Data & Safety Monitoring Board (DSMB). The second dosing scheme proceeded as follows: [0 (Saline), 0.14, 0.28, and 0.56 mg/ 70 kg], retaining the original IM route of administration. Nine participants (6 healthy and 3 CLBP) were exposed to this second dosing scheme. Adverse events (e.g., vomiting) were observed in 4 participants. After further consultation with the DSMB, we retained the same doses [0 (Saline), 0.14, 0.28, and 0.56 mg/70 kg], but changed the route of administration from IM to subcutaneous (SQ) injection. The remaining 23 participants (16 healthy and 7 CLBP) were exposed to this third dosing scheme. Under this dosing scheme, adverse events were observed in 4 participants. Unblinded investigators evaluated participant’s responses following each dose and discontinued subsequently planned doses if participants evidenced strong opioid agonist effects, which could include 1) a substantial decrease in pulse, pupil size, or blood pressure from baseline, 2) reports of strong subjective drug effects, or 3) adverse events such as vomiting. Thus, the number of cumulative doses administered varied across participants (ranging from 2 to 4 total administrations) to calibrate all participants to a similar threshold of drug tolerability and safety. Additionally, due to strong agonist effects requiring enhanced safety monitoring, three individuals had their inter-injection intervals extended to 90 min.

#### Study 2 (n = 45)

All participants received the same dosing scheme for this study, which included SQ injections of hydromorphone: [0 (Saline), 0.28, and 0.56 mg/70 kg]. Doses were separated by 60-minute inter-injection intervals. In this study, adverse events were observed in 15 participants, but the dosing regimen remained constant throughout.

### Measures

#### Subjective Visual Analog Scale (VAS) ratings

VAS ratings (0–100, where 0 = Not at All and 100 = Extremely) were obtained at each 30-minute timepoint for a variety of subjective responses to drug administration. Subjective ratings were collected every 30 min throughout the protocol. When timepoints coincided with dosing, ratings were acquired immediately prior to the dose administration. Participants completed these items on a laptop computer while seated. A research coordinator was available in the room during questionnaire completion to answer any questions. Ratings for three single VAS items (Overall “Drug Effects”, “Good Effects”, and “Bad Effects”) were used for the present analyses. We present additional measures of risk for problematic use (i.e., “drug liking”, “how high”) in Supplementary material.

#### Physiological measures

In both studies, heart rate (beats per minute) was collected at each 30-minute time point. As with subjective ratings, timepoints that coincided with dose administration were collected prior to dosing. Pupil diameter (mm) was also collected in Study 1 using a pupilometer (Neuroptics, Irvine, CA). Both heart rate and pupil diameter were selected as representative physiological measures because they are both known to dose-dependently decrease with hydromorphone relative to placebo [9, 20]. Additional vital signs (blood pressure [BP], and respiratory rate [RR]) were monitored at 30-minute intervals for safety.

### Data analysis plan

Descriptive statistics are presented as means and standard deviations for continuous variables, or frequencies and proportions for categorical variables. Participants were categorized as Opioid Non-Responders if their reported VAS ratings of Drug Effects were < 20/100 across all assessments [8], whereas participants were categorized as Opioid Responders if reported Drug Effects exceeded > 20/100 at any post-baseline assessment. We selected this 20-point cut-off to align with previous studies and enhance comparability with prior work in this area [8–10]. Drug Effects was chosen to differentiate Responders from Non-Responders because this encompassed people who had either pleasant or aversive effects [10]. Differences across categorical variables (e.g., sex, race, study, and/or Responder Status) were evaluated with Chi-square/Fisher’s Exact tests. Correlations across subjective VAS ratings of Drug Effects, Good Effects, and Bad Effects within 60 min post administration were assessed using Pearson’s correlation coefficient (*r*).

We used linear mixed effects regression models (LMERs) to evaluate the changes in VAS ratings and physiological measures up to 60 min after drug/placebo administration. The fixed effects of interest were time since drug/placebo administration (0–60 min), Responder Status (reference: Opioid Non-Responder), and Drug Condition (reference: placebo). Interactions between time and Opioid Responder Status, and time and Drug Condition were also included. Participant IDs were included as random effects to account for within-participant correlations. The analytic approach of evaluating effects serially within 60-minute post-injection bins was chosen as opposed to a global dose-response curve model because there was substantial variability across the sample in the number of doses received, as well as the dose given at each interval. This window was also applied to the small number of people who had 90-minute injection windows, standardizing the time course analyzed for each individual. The raw data were first processed such that only VAS ratings and physiological measures up to 60 min post-injection were included. Next, the data was restructured into a long format in which each row represents one measurement at each assessment time point per participant. For each participant, each 60-minute post-injection bin consists of a maximum of three time points of data (0, 30, and 60 min), resulting in a maximum of three rows per 60-minute bin per participant. The random intercept included in the LMERs accounts for potential within-participant correlations across assessment time points.

Considering that Opioid Responder Status was operationalized with VAS ratings of Drug Effects, LMERs for Drug Effects were performed only among Opioid Responders (i.e., Responder Status was not included as a fixed effect) to evaluate the extent to which subjective drug effects over time (up to 60 min after administration) differ between placebo and hydromorphone administration among Opioid Responders. This model can be represented by the following: *y(Drug Effects)*_*ij*_ = *B*_*0*_ + *B*_*1*_*(Time*_*ij*_*)* + *B*_*2*_*(Drug Condition*_*ij*_*)* + *B*_*3*_*(Time*_*ij*_ *x Drug Condition*_*ij*_*)* + *u*_*0i*_ *+ error*_*ij*_. The full sample was utilized (both Opioid Responders and Opioid Non-Responders) in the unadjusted LMERs for VAS ratings, separately for Good Effects, and Bad Effects, as well as for heart rate and pupil diameter (for Study 1 only). These models can be expressed as *y(Outcome)*_*ij*_ = *B*_*0*_ + *B*_*1*_*(Time*_*ij*_*)* + *B*_*2*_*(Responder Status*_*j*_*)* + *B*_*3*_*(Drug Condition*_*ij*_*)* + *B*_*4*_*(Time*_*ij*_ *x Responder Status*_*j*_*)* + *B*_*5*_*(Time*_*ij*_ *x Drug Condition*_*ij*_*)* + *u*_*0i*_ *+ error*_*ij*_. Similar LMERs were also performed, adjusting for sex (reference: female) and race (reference: white). Variables in the model were not mean-centered. All statistical analyses were performed in R version 4.3.0 [21] using the lme4 package [22]. Code is available upon request from the authors.

## Results

### Sample characteristics

Sample characteristics are presented in Table 1. The sample contained more female (68.3%) than male (31.7%) participants and included an approximately equal number of white and non-white participants.

**Table 1:** Demographic Information.

	Overall N (%) or M(SD)	Study 1 N (%) or M(SD)	Study 2 N (%) or M(SD)
Sex			
Female	56 (68.3%)	23 (62.2%)	33 (72.3%)
Male	26 (31.7%)	14 (37.8%)	12 (26.7%)
Age, M (SD)	32.5 (10.5)	37 (11.3)	29 (8.1)
Race			
White	45 (54.9%)	21 (56.8%)	24 (53.3%)
Black/African American	20 (24.4%)	9 (24.3%)	11 (24.4%)
Asian	11 (13.4%)	4 (10.8%)	7 (15.6%)
American Indian/Alaskan Native	3 (3.7%)	1 (2.7%)	2 (4.4%)
More than one race	3 (3.7%)	2 (5.4%)	1 (2.2%)
Ethnicity			
Hispanic/Latino	7 (8.5%)	1 (2.7%)	6 (13.3%)
Non-Hispanic/Latino	75 (91.5%)	36 (97.3%)	39 (86.7%)
Married or Partnered	21 (27.5%)	13 (36.1%)	9 (20.5%)
Employed full or part-time	52 (65.0%)	25 (69.4%)	27 (61.4%)
Income			
< $5000	4 (4.9%)	1 (2.7%)	3 (6.7%)
$5000–$11,999	4 (4.9%)	0	4 (8.9%)
$12,000–$15,999	1 (1.2%)	0	1 (2.2%)
$16,000–$24,999	6 (7.3%)	3 (8.1%)	3 (6.7%)
$25,000–$34,999	9 (11.0%)	5 (13.5%)	4 (8.9%)
$35,000–$49,999	8 (9.8%)	3 (8.1%)	5 (11.1%)
$50,000–$74,999	11 (13.4%)	7 (18.9%)	4 (8.9%)
$75,000–$99,999	8 (9.8%)	4 (10.8%)	4 (8.9%)
$100,000+	9 (29.3%)	11 (29.7%)	13 (28.9%)
Don’t know	4 (4.9%)	2 (5.4%)	2 (4.4%)
No response	3 (3.7%)	1 (2.7%)	2 (4.4%)

Participants (*N* = 82) received a median and modal cumulative dose of 0.84 mg/70 kg of hydromorphone (*M* = 0.74, SD = 0.32). The cumulative dose received did not vary by sex (*χ*^*2*^(df=5, *N* = 82) = 5.90, Cramer’s V = 0.268, Fisher’s *p* = 0.317) or race (*χ*^*2*^(df=5, *N* = 82) = 2.44, Cramer’s V = 0.173, Fisher’s *p* = 0.945). A total of 11 participants (13.4%) received two injections, 59 participants (71.9%) received three injections, and 12 participants (14.6%) received four injections. The number of injections did not differ by sex (*χ*^*2*^(df=2, *N* = 82) = 4.60, Cramer’s V = 0.237, Fisher’s *p* = 0.115) or race (*χ*^*2*^(df=2, *N* = 82) = 1.86, Cramer’s V = 0.151, Fisher’s *p* = 0.395).

### Opioid response

Across the two studies, a total of 26 individuals (31.7%) were classified as Opioid Non-Responders (i.e., reported drug effects < 20 on all VAS ratings of Drug Effects following parenteral administration of hydromorphone), whereas the remaining 56 individuals (68.3%) were classified as Opioid Responders. The proportion of participants who were Opioid Non-Responders did not differ between Study 1 (*N* = 12, 32.4%) and Study 2 (*N* = 14, 31.1%); *χ*^*2*^(df=1, *N* = 82) = 0.00, φ = 0.014, Fisher’s *p* = 1.00). Further, Opioid Responder Status did not differ by sex (*χ*^*2*^(df=1, *N* = 82) = 0.410, φ = 0.099, Fisher’s *p* = 0.447), with similar rates of male (38.5%) and female (28.6%) Opioid Non-Responders. Finally, Opioid Responder Status did not differ based on race (*χ*^*2*^(df = 1, *N* = 82) = 0.345, φ = 0.091, Fisher’s *p* = 0.557), with similar proportions of Opioid Non-Responders among individuals who identified their race as white (35.6%) or non-white (27.0%). Opioid Responder Status also did not differ by cumulative dosage (*χ*^*2*^(df=5, *N* = 82) = 1.688, φ = 0.143, Fisher’s *p* = 0.937) or route of administration (IM vs. SQ) (*χ*^*2*^(df=1, *N* = 82) = 0, φ = 0.031, Fisher’s *p* = 1.000). This suggests that differences between Opioid Responders and Opioid Non-Responders are not attributable to differences in cumulative dose or route of administration.

**Fig. 1:**
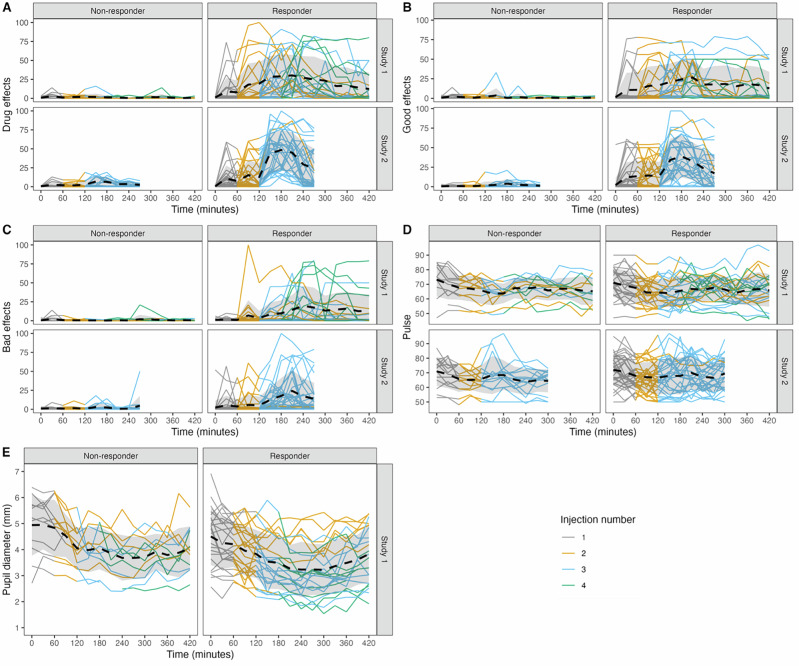
Drug Effects By Responder Status. These figures (**A**–**E**) display the slopes for each of the five outcomes (**A**. drug effects; **B**. good effects; **C**. bad effects; **D**. pulse; **E**. pupil diameter), by Responder Status and Drug Condition. The x-axis shows time since each injection. The y-axis represents predicted values, and is limited to the plausible range for each outcome.

*Among Opioid Responders*, LMERs were performed for each post-dosing window (modeled together) following drug/placebo injections. Table 2 provides complete statistics from these models. Supplementary Table 2 provides descriptive statistics for each outcome at each dose among Opioid Responders and Opioid Non-Responders. Time since last injection significantly interacted with Drug Condition (i.e., hydromorphone vs. placebo), such that the change in drug effects over time across each post-dosing window was stronger following a hydromorphone injection (relative to a placebo injection; Fig. 1A, Supplementary Fig. 1A, Supplementary Fig. 2A). Similarly, in both unadjusted and adjusted models, overall subjective Drug Effects were stronger following hydromorphone injections (relative to placebo). Sex and race were not significant covariates in the model.

**Table 2:** Subjective Drug Effects (60 minute blocks).

	Overall Drug Effects^a^		Good Drug Effects		Bad Drug Effects	
	Unadjusted Effects Estimate (95%CI)	Adjusted Effects Estimate (95%CI)	Unadjusted Effects Estimate (95%CI)	Adjusted Effects Estimate (95%CI)	Unadjusted Effects Estimate (95%CI)	Adjusted Effects Estimate (95%CI)
Intercept	1.43 (−3.99, 6.85)	0.57 (−6.61, 7.74)	−3.61 (−9.39, 2.17)	−4.38 (−11.09, 2.33)	1.00 (−2.63, 4.64)	1.05 (−3.07, 5.17)
Time since last injection (minutes)	0.25 (0.06, 0.45)*	0.25 (0.06, 0.45)*	−0.02 (−0.17, 0.13)	−0.02 (−0.17, 0.13)	−0.11 (−0.23, −0.00)*	−0.12 (−0.23, −0.00)*
Responder Status						
Opioid Non-Responder (Ref)	—	—	Ref	Ref	Ref	Ref
Opioid Responder	—	—	7.74 (1.40, 14.08)*	8.12 (1.70, 14.54)*	0.90 (−2.89, 4.69)	0.63 (−3.20, 4.46)
Drug Condition						
Placebo (Ref)	Ref	Ref	Ref	Ref	Ref	Ref
Hydromorphone	8.39 (3.99, 13.78)*	8.40 (3.01, 13.79)*	7.74 (4.20, 11.27)*	7.74 (4.20, 11.27)*	0.35 (−2.30, 3.01)	0.35 (−2.30, 3.01)
Time x Responder Status	—	—	0.33 (0.22, 0.45)*	0.33 (0.22, 0.45)*	0.22 (0.14, 0.31)*	0.22 (0.14, 0.31)*
Time x Drug Condition	0.34 (0.12, 0.55)*	0.34 (0.12, 0.55)*	0.01 (−0.13, 0.15)	0.01 (−0.13, 0.15)	0.14 (0.04, 0.25)*	0.14 (0.04, 0.25)*
Sex						
Female (Ref)	—	Ref	—	Ref	—	Ref
Male	—	0.16 (−7.95, 8.26)	—	2.85 (−3.10, 8.80)	—	−1.37 (−4.73, 1.99)
Race						
Non-white (Ref)	—	Ref	—	Ref	—	Ref
White	—	1.70 (−5.64, 9.03)	—	−0.88 (−6.45, 4.68)	—	1.27 (−1.87, 4.41)

^*^denotes *p* < 0.05.

^a^Only Opioid Responders were included in this set of models.

Overall Drug Effects, Unadjusted model: σ^2^ = 301.94, τ_00_ = 135.41, ICC = 0.31, Marginal R^2^ = 0.315, Conditional R^2^ = 0.527.

Overall Drug Effects, Adjusted model: σ^2^ = 301.84, τ_00_ = 141.84, ICC = 0.32, Marginal R^2^ = 0.313, Conditional R^2^ = 0.533.

Good Drug Effects, Unadjusted model σ^2^ = 190.09, τ_00_ = 128.86, ICC = 0.40, Marginal R^2^ = 0.237, Conditional R^2^ = 0.545.

Good Drug Effects, Adjusted model σ^2^ = 190.03, τ_00_ = 130.94, ICC = 0.41, Marginal R^2^ = 0.239, Conditional R^2^ = 0.550.

Bad Drug Effects, Unadjusted model σ^2^ = 107.51, τ_00_ = 34.53, ICC = 0.24, Marginal R^2^ = 0.160, Conditional R^2^ = 0.364.

Bad Drug Effects, Adjusted model σ^2^ = 107.46, τ_00_ = 35.03, ICC = 0.25, Marginal R^2^ = 0.164, Conditional R^2^ = 0.370.

Overall, the key findings from this set of analyses are (1) 31.7% of the sample met our criteria for Opioid Non-Responder phenotype, a rate nearly identical to prior oral hydromorphone dosing studies [8–10, 20]; (2) opioid response did not vary by dose or route of administration; 3) Opioid Responders discriminated drug from placebo in the acute post-dosing windows, and 4) there were no differences in opioid response profiles based on race or sex.

### “Good” and “Bad” drug effects

Because the original Opioid Responder group variable was created based on Drug Effects VAS ratings, it is plausible that the Opioid Non-Responder group could have demonstrated effects on other VAS ratings. To evaluate the consistency of this phenotype across different VAS response items, the LMERs were extended to evaluate Opioid Responder group differences on “Good” and “Bad” Effects, which were administered as separate VAS items. As shown in Table 2, in both unadjusted and adjusted models, Good Effects were stronger among Opioid Responders relative to Opioid Non-Responders, and respondents reported stronger Good Effects following hydromorphone relative to placebo. Further, time since last injection interacted with Opioid Responder status to predict Good Effects, such that Good Effects increased across each post-dosing time window among Opioid Responders, but not among Opioid Non-Responders (Fig. 1B, Supplementary Fig. 1B, Supplementary Fig. 2B). Sex, race, time since last injection, and the interaction between time since last injection and Drug Condition (hydromorphone vs. placebo) were not significant.

Bad Effects decreased within each post-dosing window as the time since the last injection increased. Similarly, time since injection interacted with Opioid Responder Status, such that Bad Effects tended to increase over time within each post-dosing window among Opioid Responders but not among Opioid Non-Responders. Time also interacted with Drug Condition, such that Bad Effects increased over time within each post-dosing window following hydromorphone injections but not placebo injections. When stratified by Drug Condition, the slope of Bad Effects for Opioid Responders showed greater increases following hydromorphone injections (b = 0.25, 95% CI: 0.24, 0.33), relative to placebo injections (b = 0.11, 95% CI = 0.07, 0.24). (Fig. 1C, Supplementary Fig. 1C, Supplementary Fig. 2C). There was no main effect of Responder Status, and Opioid Non-Responders did not report greater Bad Effects following hydromorphone relative to placebo. Race and sex were not significantly related to Bad Effects ratings.

As described in Supplementary Table 3, the results for “Like Drug” closely mirrored those for “Good Drug Effects,” with significant main effects of Responder Status (i.e., greater drug liking in the Opioid Responder group) and Drug Condition (i.e., greater drug liking following hydromorphone injections), as well as a Time x Responder Status interaction (i.e., drug liking increased more over time in the Opioid Responder group). For “How High,” a similar pattern emerged, with a main effect of Drug Condition and a Time x Responder Status interaction. Additionally, there was a significant Time x Drug Condition interaction, indicating that increases in ratings were greater following hydromorphone compared to placebo.

### Physiological effects

#### Heart rate

As shown in Table 3, in both unadjusted and adjusted models, heart rate was lower following hydromorphone relative to placebo injections. However, in contrast to subjective effects, heart rate was *not* predicted by Responder Status or time since last injection. Interactions between time and both Responder Status and Drug Condition were non-significant (Fig. 1D, Supplementary Fig. 1D, Supplementary Fig. 2D). Race was not associated with heart rate. There was a main effect of sex on heart rate, such that males, on average, had a lower heart rate than females.

**Table 3:** Objective Drug Effects, 60 minutes.

	Heart Rate		Pupil Diameter (Study 1 Only)	
	Unadjusted Effects Estimate (95%CI)	Adjusted Effects Estimate (95%CI)	Unadjusted Effects Estimate (95%CI)	Adjusted Effects Estimate (95%CI)
Intercept	71.06 (67.40, 74.72)^a^	72.19 (67.99, 76.39)^a^	4.99 (4.44, 5.53)^a^	5.02 (3.91, 6.12)^a^
Time since last injection (minutes)	−0.04 (−0.10, 0.01)	−0.05 (−0.10, 0.01)	−0.01 (−0.01, 0.00)	−0.01 (−0.01, 0.00)
Responder Status				
Opioid Non-Responder (Ref)	Ref	Ref	Ref	Ref
Opioid Responder	0.64 (−3.64, 4.91)	−0.12 (−4.25, 4.02)	−0.49 (01.13, 0.15)	−0.52 (−1.22, 0.18)
Drug Condition				
Placebo (Ref)	Ref	Ref	Ref	Ref
Hydromorphone	−4.95 (−6.35, −3.56)^a^	−4.95 (−6.35, −3.56)^a^	−0.46 (−0.63, −0.28)^a^	−0.46 (−0.63, −0.28)^a^
Time x Responder Status	−0.02 (−0.06, 0.03)	−0.02 (−0.06, 0.03)	−0.00 (−0.01, 0.01)	−0.00 (−0.01, 0.01)
Time x Drug Condition	0.05 (−0.00, 0.11)	0.05 (−0.00, 0.11)	−0.01 (0.01, −0.00)^a^	−0.01 (−0.01, −0.00)^a^
Sex				
Female (Ref)	—	Ref	—	Ref
Male	—	−5.25 (−9.30, −1.21)^a^	—	0.29 (−0.35, 0.93)
Race				
Non-white (Ref)	—	Ref	—	Ref
White	—	2.33 (−1.45, 6.11)	—	−0.00 (−0.02, 0.01)

^a^denotes *p* < 0.05, Adjusted Pupil diameter model also controlled for route – nonsignificant, data not shown.

Heart Rate, Unadjusted model σ^2^ = 29.52, τ_00_ = 75.34, ICC = 0.72, Marginal R^2^ = 0.035, Conditional R^2^ = 0.728.

Heart Rate, Adjusted model σ^2^ = 29.52, τ_00_ = 68.90, ICC = 0.70, Marginal R^2^ = 0.111, Conditional R^2^ = 0.733.

Pupil, Unadjusted model, σ^2^ = 0.21, τ_00_ = 0.80, ICC = 0.79, Marginal R^2^ = 0.178, Conditional R^2^ = 0.831.

Pupil, Adjusted model, σ^2^ = 0.21, τ_00_ = 0.87, ICC = 0.81, Marginal R^2^ = 0.189, Conditional R^2^ = 0.845.

#### Pupil diameter

Pupillometry was only performed in Study 1. As shown in Table 3, in both unadjusted and adjusted models, pupil diameter was smaller (consistent with opioid agonist effects) following hydromorphone relative to placebo injections. There was a Drug Condition X Time interaction in both unadjusted and adjusted models, such that decreases in pupil size across each post-dosing time window were greater following hydromorphone injections relative to placebo injections (Fig. 1E, Supplementary Fig. 1E, Supplementary Fig. 2E). Consistent with heart rate, pupil diameter effects diverged from subjective ratings and did not differ as a function of Opioid Responder Status. The adjusted model also controlled for route of administration (non-significant, not shown). Neither sex nor race was associated with pupil diameter.

Collectively, these findings indicate that all individuals were experiencing conventional physiological responses to an opioid agonist regardless of their subjective awareness on VAS ratings.

## Discussion

The purpose of these analyses was to characterize subjective and physiological responses to parenterally administered hydromorphone, relative to placebo, in persons who had no existing physical dependence or tolerance on opioids. Results suggest that cumulative, parenteral hydromorphone relative to placebo produced physiological changes (i.e., decreases in heart rate and pupil size) consistent with typical acute opioid agonist effects [23]. However, subjective opioid response varied, with 31.7% of participants reporting no meaningful drug effects across all doses and VAS rating opportunities. Notably, Opioid Non-Responders were demographically similar to Opioid Responders. This percentage is strikingly consistent with prior evaluations of this effect following oral opioid administration [8–10, 20], which also suggests that approximately 30% of participants without a history of prolonged or problematic opioid use who received therapeutic doses of orally administered hydromorphone do not endorse drug effects despite exhibiting physiological responses [10]. The extension of these findings to cumulative parenteral opioid administration is important and novel because it demonstrates for the first time that the Opioid Non-Responder phenotype is independent of first-pass P450 metabolism [11], and is evident when escalating opioid doses are administered repeatedly in close succession. Taken together with previous findings from oral administration studies, the present results strongly indicate the presence of an underlying phenotype characterized by reduced subjective sensitivity to opioids in approximately 30% of the population [8–10, 20].

An estimated 11.9% of United States adults received a prescribed opioid in the past year in 2019 and 2020 [24], equating to approximately 30 million individuals. However, only a small subset (i.e., fewer than 10%) of individuals develop OUD [25, 26], suggesting that there are individual differences in OUD risk versus protection. Identifying reliable risk or protection-promoting phenotypes related to opioid response is paramount to developing early intervention strategies and/or patient selection guidance. Emerging data suggest that subjective drug experience may serve as a clinically useful tool. This is supported by prospective and retrospective evidence linking stronger subjective drug effects, particularly stimulation and euphoria, to increased risk of nonprescribed opioid use and development of OUD [27–29]. Notably, these studies have not controlled for opioid dose during initial use. In terms of protection, it is possible that individuals who fail to detect an opioid at escalating doses may be less likely to develop motivation for continued use or opioid seeking behaviors. In terms of risk, it is also plausible that individuals with low subjective awareness of opioid effects might request and receive higher doses of opioids during clinical care due to their perception of weak effects, thereby increasing their risk of opioid-induced adverse effects, including constipation, sleep disorder breathing, hyperalgesia, tolerance, physical dependence, and possibly OUD [20, 30]. This interpretation aligns with findings from the alcohol use disorder (AUD) literature, which show that young adults who report low levels of subjective intoxication to alcohol are more likely to engage in heavy alcohol use earlier in life and face an increased risk of developing AUD in adulthood, compared to peers who experience more typical alcohol effects during youth [31–33]. However, in AUD, high-risk groups also display diminished physiological responses to alcohol, such as static ataxia (i.e., body sway), hormone response (i.e., cortisol), heart rate, pulse amplitude, and skin temperature [32, 34, 35]. This dissociation between subjective and physiological effects for present opioid data raises critical questions about whether isolated low subjective opioid sensitivity represents a protective factor, by reducing the reinforcing effects of opioids and thus diminishing the desire for repeated use, or a risk factor, by promoting dose escalation due to impaired interoception and increasing the likelihood of adverse effects. Further research is needed to clarify the clinical implications of this “Opioid Non-Responder” phenotype, which has now been replicated across multiple opioid types, routes of administration, dosing schemes, and research groups, particularly in opioid-naïve patients prescribed opioids for acute pain, and with a greater range of physiological measures.

These data converge with prior reports and provide additional reliable evidence of consistent inter-individual differences in opioid response that exist prior to opioid tolerance and independent of opioid requirements for pain management [8–10, 20]. More work is now needed to identify the mechanisms underlying opioid sensitivity. The present study suggested Opioid Non-Responders did not differ from other individuals with regard to sex and race. However, this group might be meaningfully different in other important ways. For example, it is possible that this group has impaired interoceptive ability or reduced ability to perceive internal bodily cues. Altered interoceptive ability has been linked to a range of psychiatric conditions that occur at higher rates among persons with OUD, including major depressive disorder, generalized anxiety disorder, and post-traumatic stress disorder [36]. Additionally, Opioid Non-Responders may have distinct biological features, such as genetic polymorphisms affecting opioid metabolism (e.g., CYP2D6) [11, 13, 14, 37] that were not assessed in the parent studies. Limited evidence suggests that attenuated responses to opioids may be related to variation in the OPRM1 gene. Specifically, persons with the AG/GG minor genotype on the A118G SNP of the OPRM1 gene who were administered a moderate dose of oral hydromorphone reported mild, generally pleasant subject effects relative to AA-carriers [20]. In a subsequent analysis, AG/GG-carriers reported significantly higher liking for morphine relative to naltrexone and placebo, whereas AA-carriers reported similar mean ratings across drugs [38]. In addition to genetic factors, psychosocial factors, such as exposure to trauma or chronic stress, may contribute to blunted processing of interoceptive or drug-related cues [39, 40]. Alternatively, trauma exposure could sensitize individuals to opioid effects, leading to heightened responses [41]. Finally, future work should test whether Opioid Non-Responders also show reduced sensitivity to other substances. If Opioid Non-Responders show reduced sensitivity to other substances in addition to opioids, this could suggest there are common mechanisms underlying low sensitivity to reinforcing substances (e.g., low interoceptive ability) versus opioid-specific mechanisms.

Interestingly, when reporting on “Good Effects,” Opioid Responders exhibited regression coefficients of similar magnitude following both hydromorphone and placebo injections. This suggests that this subgroup demonstrated a measurable placebo response to non-active injections. In contrast, this effect was not observed among Opioid Non-Responders. Prior research has proposed that placebo effects may be mediated by the endogenous opioid system [42, 43], and are sensitive to individual differences. For example, individuals who are more responsive to placebo tend to report lower emotional distress and less pain-related catastrophizing than persons without prominent placebo responses [44]. This provides further support that more work is needed to characterize Opioid Responders and Opioid Non-Responders; these studies should incorporate assessments of emotional distress.

The results of this study should be interpreted in the context of limitations and methodological considerations. This study included healthy volunteers with or without chronic low back pain; more work needs to explore this phenomenon among persons with a known OUD, as it is not known whether the Opioid Non-Responders in this study will develop future opioid-related problems. Further, because most participants in this study were healthy volunteers, the dosing regimen in this study is not analogous to cumulative dosing in clinical settings. This study also excluded individuals with comorbidities that are more prevalent among persons with substance use disorders than healthy controls (e.g., persons with major psychiatric conditions). Additionally, because one of these studies recruited a subset of persons with chronic low back pain, individuals with pain may be over-represented in this sample, though removing these individuals in a sensitivity analysis did not impact the results. Although we did not observe a sex difference in this study, results should be replicated in larger samples, and other factors (i.e., hormonal status) should be considered. The dosing paradigm changed during Study 1, which may limit the comparability of responses across participants. Pupillometry data were only collected in Study 1. It is possible that a small number of participants who were discontinued after only one or two doses might have experienced subjective effects with additional dosing. However, we cannot fully assess this potential, as dosing was discontinued for safety reasons when objective signs of agonist effects (e.g., pupil changes) were observed. Finally, these studies tested responses to hydromorphone, which has a higher potency than oxycodone and morphine but a lower potency than fentanyl [23]. Future work may consider opioid responses among a broader range of opioids.

## Conclusion

Results from these analyses provide additional support for a phenotype characterized by attenuated subjective opioid response [8–10, 20], despite demonstrating physiological effects to opioids administered in a cumulative, parenteral dosing paradigm. This phenotype appears to be present in as many as one in three individuals, and the present results extend prior work to suggest stability in this proportion across routes of administration. Extension of this finding from oral dosing to a cumulative, parenteral dosing paradigm suggests that this effect is independent of liver metabolism and should be investigated further in acute pain management contexts. Persons who are unable to subjectively detect opioid effects may either be at a greater risk to rapidly escalate their opioid dose and develop increased tolerance and more severe withdrawal, or may be protected against such outcomes due to the absence of drug liking. These are empirical questions worthy of exploration. Future work in this area could seek to find ways to track the emergence or absence of opioid related problems and OUD among Opioid Non-Responders and identify factors and characteristics that occur at a higher rate among such Opioid Non-Responders.

## Supplementary information

Supplemental Material

## Data Availability

Data are available upon request.
