# Supplementary material for: Reliable variability in subjective responses to parenteral hydromorphone administration: empirical confirmation of an opioid non-responder phenotype
Source: Neuropsychopharmacology. 2025 Nov 18;51(2):455–63. doi: 10.1038/s41386-025-02271-4 (PMC12708843; doi:10.1038/s41386-025-02271-4)

Supplemental Material

Study 1: Both healthy participants (N=27) and patients with chronic low back pain (CLBP; N=10) were recruited into this study. Inclusion and exclusion criteria are described for the full Study 1 sample and for each group specifically below:

General Inclusion Criteria. 1) 18-60 year olds; 2) less than 2 servings/day of caffeinated beverages or willing to discontinue 3 days prior to admission; 3) any prior history of opioid use.

CLBP-Specific Inclusion Criteria. In addition to the general inclusion criteria, participants in the CLBP group had to have: 1) a physician-confirmed diagnosis of CLBP; 2) report low back pain on average at least 3/10 pain intensity on a majority of days over the prior 3 months.

General Exclusion Criteria. 1) BMI >40; 2) significant medical or psychiatric morbidity within 6 months of study enrollment or a lifetime history of bipolar disorder, psychotic disorder, seizure disorder, opioid use disorder; 3) clinically significant abnormal complete blood count or comprehensive metabolic profile; 4) current or recent (prior 6 months) renal or hepatic disease, or obstructive sleep apnea; 5) current use of opioids or benzodiazepines for any purpose; 6) positive toxicology screen for opioids, stimulants, or recreational drugs; 7) pregnancy or lactation; 8) significant; 9) previous adverse reactions to opioids; 10) serious or significant adverse events associated with any dose, including placebo; 11) A QTc interval of more than 450msec or clinically significant ECG findings. 12) Any medication, medical condition or behavior observed during participation that the investigators determined could compromise the integrity or safety of the study.

Healthy Control and CLBP-Specific Exclusion Criteria. In addition to the general exclusion criteria, healthy controls were excluded if they reported a current medical/psychiatry history not explicitly specified above but that was counter-indicated with participation; reported an acute painful injury (within 3 months); had a diagnosed chronic pain disorder (i.e., healthy controls could not have a chronic pain disorder). CLBP participants were excluded for the presence of any comorbid idiopathic or rheumatic chronic pain condition.

Study 2: Healthy adults (N=45) were recruited into this study.

Inclusion Criteria: 1) Healthy, 18-48 year olds meeting Research Diagnostic Criteria for Normal Sleepers; 2) a stable sleep phase, based upon PI assessment; 3) total sleep time >6.5 and <8.5 hours/night and sleep efficiency >85%, assessed using a 7-day sleep diary; 4) Pittsburgh Sleep Quality Index <5; 5) low caffeine use (the equivalent of < 3 cups, q.d.)

Exclusion Criteria: 1) BMI>40; 2) significant medical or psychiatric morbidity within 6 months or lifetime history of bipolar disorder, psychotic disorder, or seizure disorder; 3) positive toxicology screen for opioids or recreational drugs; 4) pregnancy or lactation; 5) significant preadmission psychological distress (T-scores > 64 on the Brief Symptom Inventory global scales); 6) lifetime history of chronic pain (>3 months; graded chronic pain scale > 2); 7) acute pain; 8) meeting ICSD criteria for a sleep disorder, significant breathing disruptions (AHI > 15), or frequent periodic limb movements (> 15/hour), as measured by the WatchPat HST or a PSG; 9) significant CNS disease (e.g., systemic lupus erythematosus, multiple sclerosis); 10) cognitive impairment (based on a Mini Mental State Examination, using education-adjusted cutoffs), brain injury, or history of closed head injury with loss of consciousness for greater than 3 mins; 11) use in the last 3 months of antidepressants, neuroleptics, sedative hypnotics, isoniazid,

glucocorticoids, psychostimulants, or opioids; 12) respiratory, hepatic, renal, or cardiac conditions that would contraindicate opioid use; 13) lifetime history of an opioid use disorder; 14) prior adverse reaction to general anesthetics/opioids, unable to tolerate 0.28 mg/70 kg hydromorphone; 15) clinically significant abnormal ECG, complete blood count, hepatic, renal, or metabolic panel; 16) embedded metal objects/fragments or electronic devices in the head or body that would present a risk during an MRI; 17) exposure to ionizing radiation that, combined with the primary study's estimated exposure, would exceed recommended limits; 18) inability to tolerate a scanning environment/claustrophobia; 19) resting heart rate < 60 bpm; 20) regular smokers unwilling/unable to remain smoke-free during study participation.

Supplemental Table 1. Subjective drug effects, Excluding persons with CLBP

	Overall Drug Effects[1]		Good Drug Effects		Bad Drug Effects	
	Unadjusted Effects Estimate (95%CI)	Adjusted Effects Estimate (95%CI)	Unadjusted Effects Estimate (95%CI)	Adjusted Effects Estimate (95%CI)	Unadjusted Effects Estimate (95%CI)	Adjusted Effects Estimate (95%CI)
Intercept	1.46 (-4.33, 7.33)	0.58 (-7.46, 8.62)	-3.11 (-9.39, 2.17)	-3.46 (-10.00, 3.07)	0.97 (-2.90, 4.83)	0.80 (-3.55, 5.16)
Time since last injection (minutes)	0.24 (0.03, 0.45)*	0.24 (0.03, 0.45)*	-0.03 (-0.19, 0.12)	-0.03 (-0.19, 0.12)	-0.11 (-0.23, 0.01)	-0.11 (-0.23, 0.01)
Responder Status						
Opioid Non-Responder (Ref)	-	-	Ref	Ref	Ref	Ref
Opioid Responder	-	-	7.31 (1.07, 13.55)*	7.64 (1.30, 13.97)*	1.23 (-2.86, 5.31)	0.89 (-3.24, 5.03)
Drug Condition						
Placebo (Ref)	Ref	Ref	Ref	Ref	Ref	Ref
Hydromorphone	8.66 (2.85, 14.47)*	8.67 (2.86, 14.48)*	7.00 (3.32, 10.69)*	7.01 (3.32, 10.69)*	0.34 (-2.49, 3.18)	0.34 (-2.49, 3.17)
Time x Responder Status	-	-	0.31 (0.19, 0.43)*	0.31 (0.19, 0.43)*	0.22 (0.13, 0.31)*	0.22 (0.13, 0.31)*
Time x Drug Condition	0.35 (0.12, 0.57)*	0.34 (0.12, 0.57)*	0.03 (-0.12, 0.18)	0.03 (-0.12, 0.18)	0.14 (0.03, 0.25)*	0.14 (0.03, 0.25)*
Sex						
Female (Ref)	-	Ref	-	Ref	-	Ref
Male	-	0.29 (-8.79, 9.38)	-	2.41 (-3.63, 8.44)	-	-1.37 (-5.15, 2.41)
Race						
Non-white (Ref)	-	Ref	-	Ref	-	Ref
White	-	1.54 (-6.56, 9.64)	-	-1.22 (-6.76, 4.32)	-	1.67 (-1.80, 5.14)

Supplemental Table 2. Descriptive statistics of outcome variables by injection number, study, cumulative dosage, and responder status.

			Non-responders				Responders			
		Injection number	1	2	3	4	1	2	3	4
Outcome	Study	Cumulative dosage	M (SD)	M (SD)	M (SD)	M (SD)	M (SD)	M (SD)	M (SD)	M (SD)
Drug effects	1	0.14	2.88 (4.73)	0.43 (0.94)			18.50 (25.63)	21.65 (27.35)		
	1	0.33					12.33 (6.43)	23.09 (23.17)		
	1	0.42	1.00 (1.80)	0.33 (0.71)	1.33 (3.24)		0.61 (0.98)	11.22 (21.38)	22.87 (28.75)	
	1	0.98	1.25 (2.82)	2.62 (2.39)	1.00 (1.60)	1.64 (2.66)	1.75 (1.21)	4.40 (9.86)	18.20 (26.44)	23.71 (26.40)
	1	2.28					0 (0)	0 (0)	13.50 (19.09)	48.56 (15.77)
	2	0.84	1.61 (3.17)	1.93 (2.89)	4.26 (5.12)		5.76 (13.13)	15.28 (21.87)	33.18 (28.53)	
Good effects	1	0.14	2.62 (4.81)	0.31 (0.76)			24.30 (30.93)	23.18 (24.59)		
	1	0.33					3.67 (4.73)	5.91 (11.10)		
	1	0.42	0.89 (1.36)	0.44 (1.01)	1.95 (5.80)		1.28 (1.53)	10.72 (23.25)	28.66 (28.82)	
	1	0.98	1.12 (2.10)	2.00 (2.33)	1.38 (1.92)	1.06 (1.24)	1.65 (1.31)	4.25 (8.88)	13.18 (16.02)	12.63 (18.21)
	1	2.28					0 (0)	0 (0)	6.00 (8.49)	13.56 (8.96)
	2	0.84	0.79 (1.26)	0.82 (1.70)	2.27 (4.08)		7.18 (15.87)	17.57 (23.07)	25.83 (24.94)	
Bad effects	1	0.14	2.50 (4.84)	0.33 (0.74)			3.20 (3.19)	10.08 (19.55)		
	1	0.33					0 (0)	7.54 (8.09)		
	1	0.42	0.44 (1.33)	0.44 (0.73)	0.45(0.80)		1.06 (1.55)	3.06 (3.99)	11.68 (20.34)	
	1	0.98	1.25 (2.55)	1.50 (2.51)	0.62 (1.19)	2.08 (4.15)	1.10 (0.91)	1.70 (1.72)	6.36 (9.68)	19.21 (26.45)
	1	2.28					0 (0)	0 (0)	6.00 (8.49)	49.78 (18.15)
	2	0.84	1.07 (1.84)	1.11 (2.33)	1.98 (6.07)		3.76 (11.11)	4.53 (11.22)	15.41 (22.05)	
Pulse	1	0.14	64.75 (7.63)	63.31 (7.35)			69 (7.73)	66.63 (8.11)		
	1	0.33					62.33 (5.03)	65.91 (6.89)		
	1	0.42	79.78 (5.04)	73.56 (2.79)	70.38 (6.04)		70.89 (11.47)	64.45 (10.04)	66.43 (11.86)	
	1	0.98	68.38 (13.62)	64.62 (9.61)	63.38 (8.85)	64.67 (7.82)	69.90 (10.68)	66.05 (9.99)	63.00 (8.24)	65.16 (9.25)
	1	2.28					76.00 (4.24)	72.00 (1.41)	67.50 (3.54)	71.22 (7.66)
	2	0.84	70.00 (9.68)	65.36 (7.77)	65.86 (9.52)		71.32 (10.72)	68.54 (9.68)	67.59 (11.33)	
Pupil[1]	1	0.14	5.14 (0.84)	4.37 (0.78)			5.34 (0.72)	4.64 (0.51)		
	1	0.33					5.41 (0.04)	4.65 (0.51)		
	1	0.42	4.51 (0.97)	4.59 (0.92)	3.71 (0.80)		3.98 (1.00)	3.68 (0.96)	3.22 (0.89)	
	1	0.98	5.11 (1.19)	4.62 (1.10)	3.80 (0.77)	3.56 (0.71)	4.12 (0.99)	3.79 (0.82)	3.12 (0.81)	2.91 (0.81)
	1	2.28					4.70 (0.11)	4.05 (0.46)	3.50 (0.13)	2.71 (0.19)

[1]Pupil diameter was not assessed in study 2.

Supplemental Table 3. Subjective Effects (60 minute blocks)

	Like drug		How high	
	Unadjusted Effects Estimate (95%CI)	Adjusted Effects Estimate (95%CI)	Unadjusted Effects Estimate (95%CI)	Adjusted Effects Estimate (95%CI)
Intercept	-2.78 (-8.74, 3.18)	-3.84 (-10.77, 3.100	-0.75 (-5.62, 4.12)	-1.47 (-7.09, 4.15)
Time since last injection (minutes)	0.06 (-0.10, 0.22)	0.06 (-0.10, 0.22)	-0.13 (-0.26, 0.01)	-0.12 (-0.26, 0.01)
Responder Status				
Opioid Non-Responder (Ref)	Ref	Ref	Ref	Ref
Opioid Responder	8.59 (2.07, 15.12)*	8.61 (1.98, 15.24)*	3.15 (-2.09, 8.38)	3.18 (-2.14, 8.50)
Drug Condition				
Placebo (Ref)	Ref	Ref	Ref	Ref
Hydromorphone	6.94 (3.27, 10.61)*	6.94 (3.27, 10.61)*	3.32 (0.09, 6.56)*	3.32 (0.09, 6.56)*
Time x Responder Status	0.27 (0.15, 0.39)*	0.27 (0.15, 0.39)*	0.36 (0.26, 0.47)*	0.36 (0.26, 0.47)*
Time x Drug Condition	-0.07 (-0.22, 0.07)	-0.07 (-0.22, 0.07)	0.15 (0.02, 0.28)*	0.15 (0.02, 0.28)*
Gender				
Female (Ref)	-	Ref	-	Ref
Male	-	1.39 (-4.75, 7.52)	-	1.03 (-3.79, 5.86)
Race				
Non-white (Ref)	-	Ref	-	Ref
White	-	1.35 (-4.39, 7.08)	-	0.80 (-3.71, 5.32)

*denotes p<.05

Like drug, Unadjusted model: σ^2= 205.11, τ_{00}= 135.36, ICC=0.40, Marginal R^2 = 0.188, Conditional R^2 = 0.511

Like drug, Adjusted model: σ^2= 205.08, τ_{00}= 139.01, ICC=0.40, Marginal R^2 = 0.188, Conditional R^2 = 0.516

How high, Unadjusted model σ^2= 159.39, τ_{00}= 79.05, ICC=0.33, Marginal R^2 = 0.252, Conditional R^2 = 0.500

How high, Adjusted model σ^2= 159.37, τ_{00}= 81.39, ICC=0.34, Marginal R^2 = 0.251, Conditional R^2 = 0.504

Supplemental Figure 1.

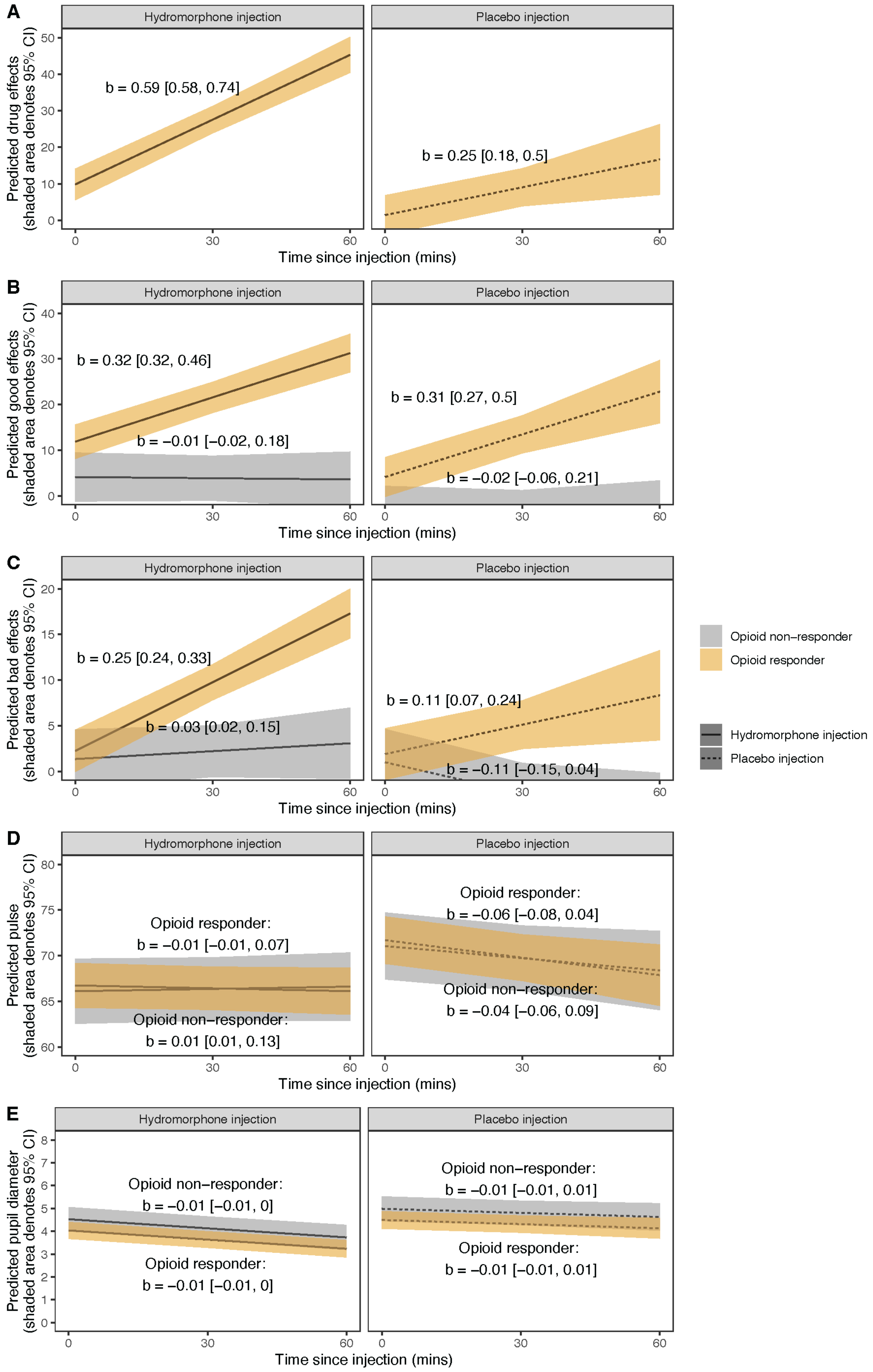

Figure Legend: These figures display the slopes for each of the five outcomes (i.e., predicted drug effects, predicted good effects, predicted bad effects, pulse, pupil diameter), by Responder Status and Drug Condition. The x-axis shows time since each injection. The y-axis represents predicted values, and is limited to the plausible range for each outcome.

Supplemental Figure 2.

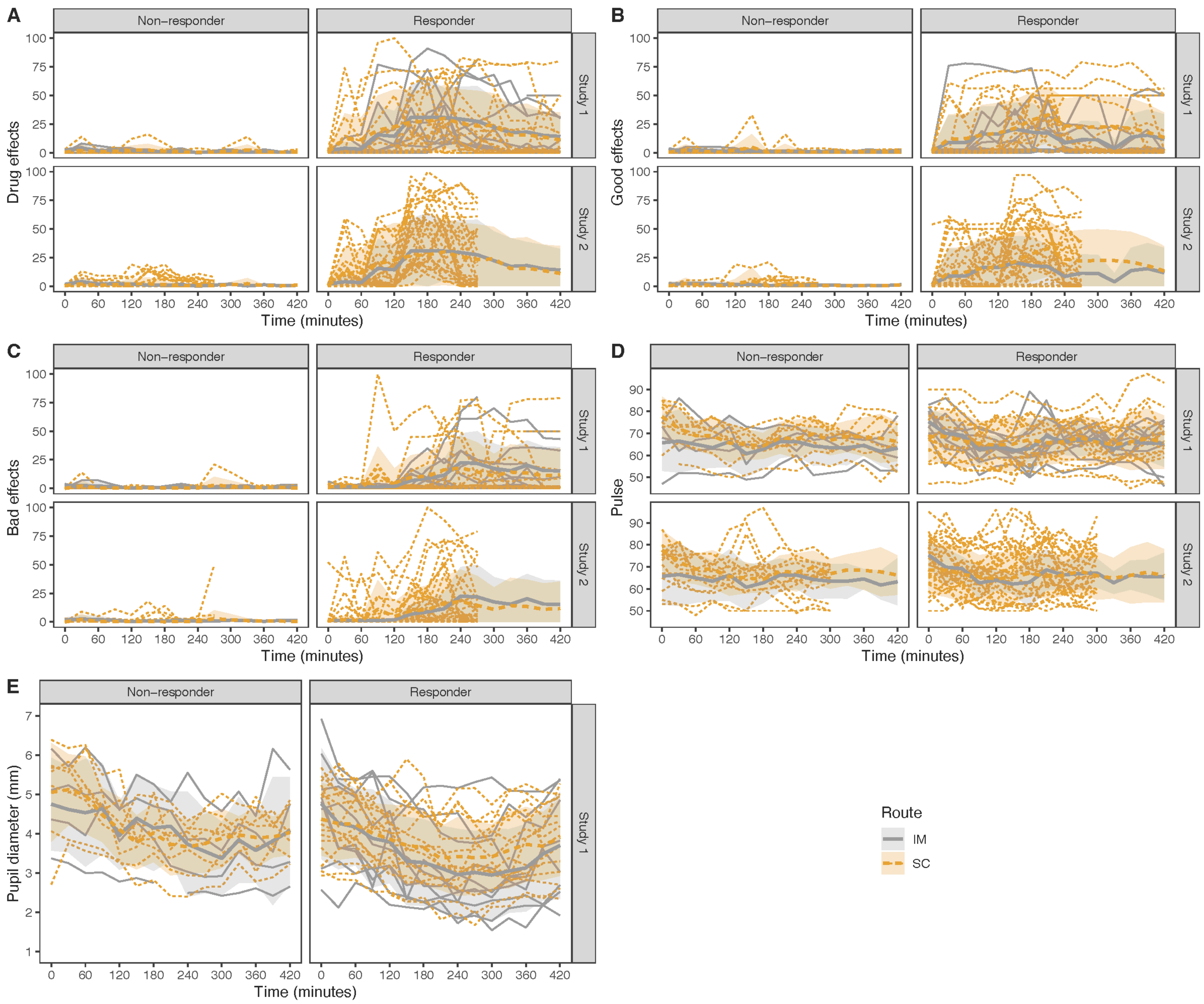

Supplement: Supplementary file 1 — Supplemental Material [file 41386_2025_2271_MOESM1_ESM.pdf]
